# Gel-Grown Kebab-like KDP Crystal Formation Mechanisms

**DOI:** 10.3390/molecules31101744

**Published:** 2026-05-20

**Authors:** Fugui Cai, Jie Ren, Yuqing Yao, Hanying Li

**Affiliations:** Ministry of Education Key Laboratory of Macromolecular Synthesis and Functionalization, International Research Center for X Polymers, Department of Polymer Science and Engineering, Zhejiang University, Hangzhou 310027, China; fgcai@zju.edu.cn (F.C.); renjie0917@outlook.com (J.R.)

**Keywords:** potassium dihydrogen phosphate (KDP), gel-grown crystal, crystallization, morphology control

## Abstract

Using the gel-grown method to control the morphology of crystals attracts extensive attention. Potassium dihydrogen phosphate (KDP) is a nonlinear optical crystal with a high laser damage threshold. Here, we studied the crystallization of KDP in silica gel. The kebab-like KDP crystals (multiple KDP crystals aligning along a straight line) were prepared in the silica gel. In situ observation revealed that the kebab-like crystals were obtained through secondary nucleations on preformed needle-like crystals. Further investigation revealed that the hydroxyl groups on the gel network have an important influence on the formation of kebab-like KDP crystals. The hydroxyl groups on the gel networks can form hydrogen bonds with the phosphoric acid group of the KDP crystal and hinder the growth of the prismatic KDP faces, which leads to the preformation of needle-like crystals. Additionally, the influence of the acetic acid concentration and antisolvent on morphology was also studied.

## 1. Introduction

Growing crystals in the gel network (gel-grown method) is an efficient method for preparing high-quality single crystals, such as high-quality protein single crystals [1,2,3,4]. During the crystallization process, the gel network can be incorporated into the gel-grown crystal, which can help to enhance the ability of morphology control [5,6,7,8,9,10,11]. Additionally, the diffusion of substances in the gel is confined, which also helps to precisely control the crystallization process and crystal morphology [8,9]. Therefore, the gel-grown method is capable of preparing high-quality single crystals and enabling precise control over their morphology.

**Figure 1:**
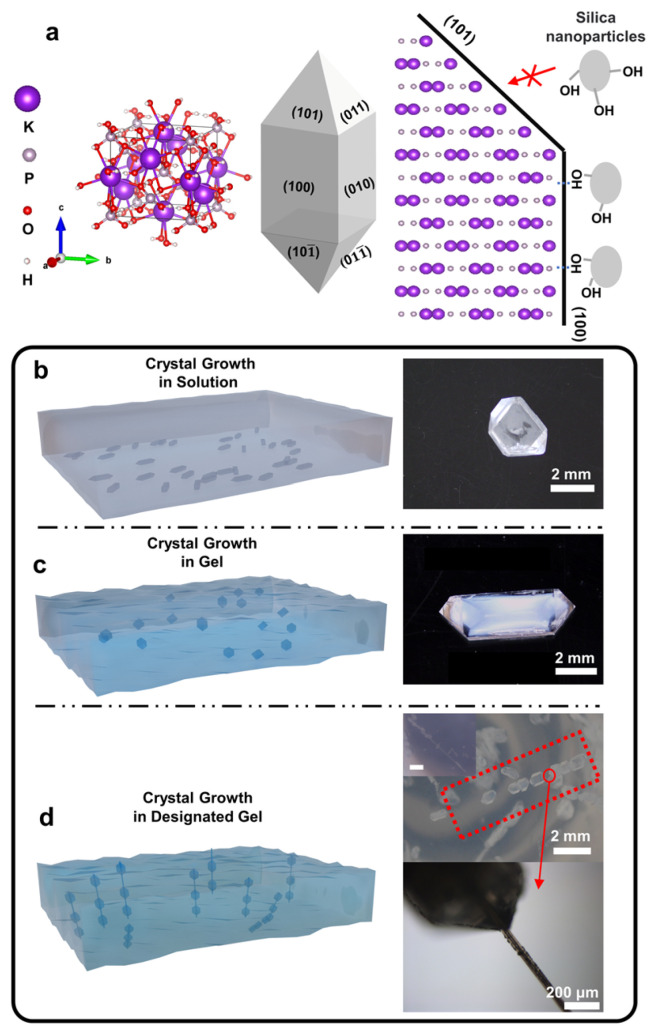
(**a**) The unit cell of the KDP crystal, the atoms of the prismatic {100} facets of the KDP crystal, and the atoms of the pyramidal {101} facets of the KDP crystal. Schematic diagram of the effect of silica gel on KDP crystals. Oxygen and hydrogen atoms are removed for simplification. (**b**–**d**) Schematic and OM images of crystal growth in solution, gel, and designated gel.

Potassium dihydrogen phosphate (KDP) is a nonlinear optical crystal with a high laser damage threshold. It is widely used in frequency doubling, electro-optic modulation, and high-power laser systems [12,13,14,15]. The prism of the typical KDP crystal is formed by the {100} facets. The phosphate groups and potassium ions are distributed on the {100} facets alternately (Figure 1a) [16]. The previous studies on the morphology control of KDP crystals are mostly conducted in solution and focused on the influence of trace ions [17,18,19,20] such as Fe^3+^ [19], Al^3+^ [17], Cr^3+^ [21], organic molecules [22,23,24,25,26], and nanoparticles [27,28]. Differently, during the gel-grown crystallization, the groups (e.g., hydroxyl group) on the gel network can intensely affect the crystal growth and adjust the crystal morphology [5].

Currently, the research on growth behaviors of the KDP crystals in gel matrices remains in the early stage. Gel matrices provide a confined environment that can significantly influence crystal growth kinetics, thereby facilitating the formation of novel crystalline structures with different optical properties [29,30,31]. As optical devices miniaturize, the demand for materials that integrate optimal optical and structural characteristics has increased [32,33]. However, most studies concentrated on bulk KDP crystallization. In this work, we studied the crystallization of KDP in silica gel. Interestingly, the gel-grown KDP crystals grow into kebab-like crystals (serial nucleation and epitaxial growth), which are continuously arranged along a straight line. This phenomenon is different from the previous KDP crystallization in solutions or other gel media [29,31,34,35,36,37].

## 2. Results

**Figure 2:**
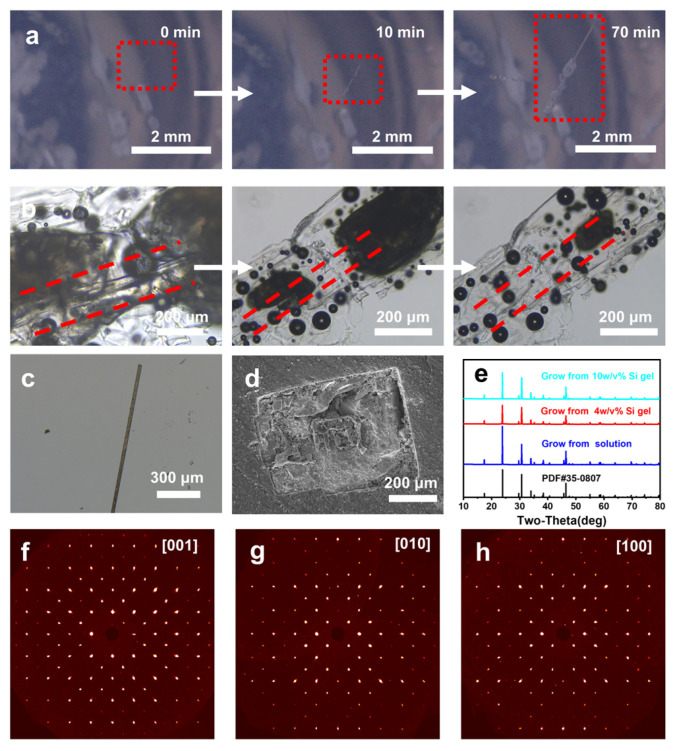
(**a**) The growth process of kebab-like KDP crystals in gel media. (**b**) The dissolution and etching process of kebab-like KDP crystals, and the red dotted line is the dissolution and etching process of inner needle-like crystals. (**c**) Needle-like crystals grown in gel medium. (**d**) SEM images of cross-sections of kebab-like KDP crystals. (**e**) PXRD patterns of KDP crystals grown at different gel concentrations and the standard tetragonal KDP phase (PDF # 35-0807). (**f**–**h**) SCXRD spot patterns of a needle-like KDP single crystal viewed from the c-axis (**f**), b-axis (**g**), and a-axis (**h**).

The gel-grown KDP crystals were grown in silica gel by diffusing methanol into gelled KDP solutions. In the silica gel with more hydroxyl groups on gel networks, the multiple gel-grown KDP crystals line up and exhibit similar crystal shapes, which look like kebabs in shape (Figure 1). The growth process of the kebab-like KDP crystals was observed in situ. Firstly, a needle-like KDP crystal with a high aspect ratio was grown from silica gel, and then multiple secondary nucleations occurred on its surface and grew into a row of crystals (Figure 2a). This growth behavior suggested a relationship with both “serial nucleation” and “epitaxial overgrowth”, reflecting the intricate interactions with the silica gel environment.

The etching experiments of the kebab-like KDP crystals were carried out using water. As the kebab-like crystals gradually dissolved, the gels similar in shape to the original crystals remained (Figure 2b), indicating the incorporation of the silica gels inside the entire crystals. Importantly, the interface between the needle crystals and the external crystals could be observed in the remaining gels, further supporting the notion that the kebab-like crystals were formed through secondary nucleation and growth on the surfaces of preformed needle-like crystals. High-aspect-ratio KDP single crystals (needle-like KDP crystals) without shell KDP were also observed in the same silica gel (Figure 2c). In addition, as imaged by a scanning electron microscope (SEM), the interface of the needle-like preformed KDP crystal and the shell KDP crystal could be observed on the cross-section of the kebab-like KDP structure (Figure 2d).

The powder X-ray diffraction (PXRD) patterns demonstrate that the kebab-like KDP crystals are in the tetragonal KDP phase, showing no shift or splitting of diffraction peaks across all crystal states (Figure 2e). The single-crystal electron diffraction spots of the internal needle-like crystals are symmetrically arranged along the [001] direction and the [100] and [010] directions (Figure 2f–h). This observation indicates that the incorporation of the silica gel network does not alter the single-crystallinity of the needle-like crystals, which still maintain their long-range order.

**Figure 3:**
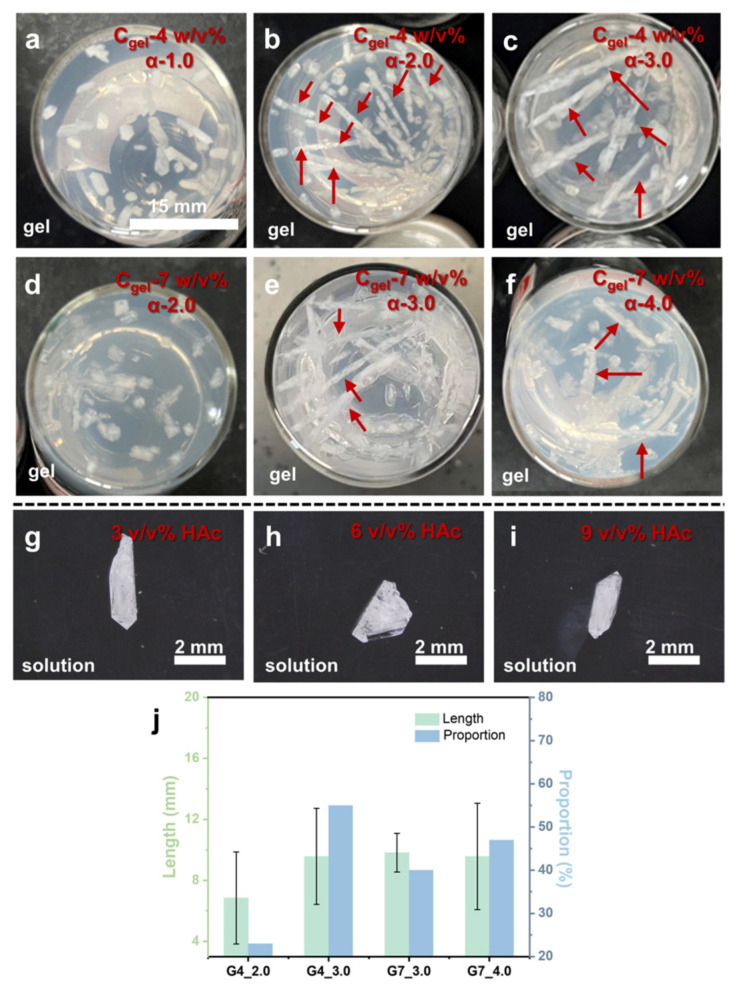
(**a**–**f**) The growth of KDP crystals in gel media with different gel concentrations and acetic acid concentrations. α is the relative addition of acetic acid to sodium metasilicate, and when α = 1, the acetic acid content is twice the molar amount of sodium metasilicate. The specific formula is shown in Table 1. The arrow points to the kebab-like crystals. Crystal growth in (**g**) 3 *v*/*v*%, (**h**) 6 *v*/*v*%, (**i**) 9 *v*/*v*% Hac, and 10 *w*/*v*% KDP solution by diffusing antisolvent methanol. (**j**) The fraction of kebab-like crystals relative to the total number of crystals and the average needle length.

Obviously, the preformation of the needle-like KDP crystal is the critical factor in forming the kebab-like KDP crystal. Nevertheless, further experiments revealed more growth mechanisms of kebab-like KDP crystals in silica gel. As shown in Figure 3a–f,j, and Table 1, the results showed that kebab-like crystals tend to grow under higher acetic acid (HAc) concentrations. Moreover, as shown in Figure 3j, with the increase in acetic acid concentration, both the probability and length of the appearing kebab-like crystals increased. Differently, in solution systems, higher HAc concentration cannot lead to the formation of kebab-like KDP crystals (Figure 3g–i).

### 2.1. The Influence of Gel Medium

In the silica gel with high HAc concentration, the growth rate of the KDP crystal pyramidal plane was much faster than the growth rate of columns, which led to the formation of a high-aspect-ratio KDP crystal (needle-like crystal). Subsequently, secondary nucleation and growth on the needle-like crystal formed the kebab-like structure. However, in the solution system, despite the use of the same concentration condition (KDP and HAc) as in the gel medium, no kebab-like KDP crystal appeared (Figure 3g–i). Therefore, the presence of silica gel has an important influence on the formation of the kebab-like KDP crystal.

In the solution, the addition of specific ions [17,18,19,20,21], organic molecules [22,23,24,25,26], or nanoparticles can inhibit the growth of some crystal faces. It is a so-called “dead zone” phenomenon in KDP crystal growth. The appearance of the growth “dead zone” is related to the anisotropic hydrogen bond skeleton in the KDP crystal, which finally determines the KDP crystal morphology (Figure 1a) [38,39,40]. In the silica gel medium, the hydroxyl groups on the gel networks can interact with the phosphate groups on the prismatic surface, which leads to an effect similar to an “impurity stopper” [19]. Compared with the prismatic surface, phosphate groups and potassium ions distribute on the pyramidal surface layer by layer, and the ends of the pyramidal surface tend to be potassium ions [16,41]. Hydroxyl groups cannot form the same effects on pyramidal surfaces as on prismatic surfaces. The distinguished difference between the growth rates of the prismatic and pyramidal crystal faces leads to the formation of a high-aspect-ratio KDP crystal (needle-like crystal) in the silica gel.

**Figure 4:**
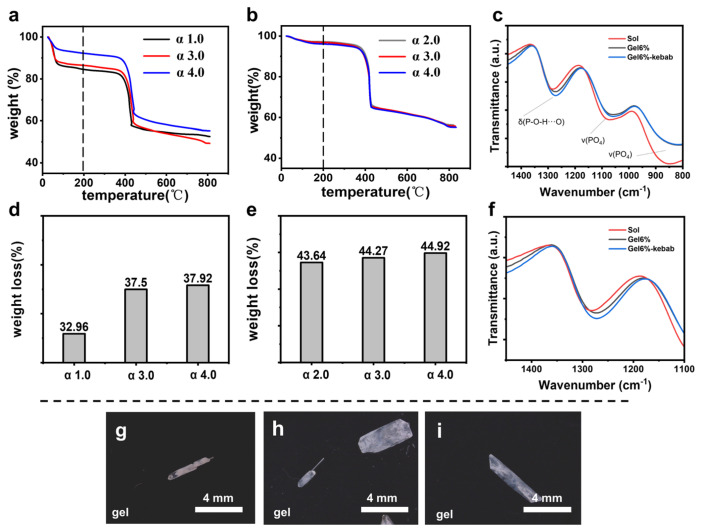
Thermogravimetric analysis (TGA) curves of different acetic acid additions at (**a**) 8 *w*/*v*% gel concentration and (**b**) 6 *w*/*v*% gel concentration. Thermal weight loss above 200 °C of different acetic acid additions at (**d**) 8 *w*/*v*% and (**e**) 6 *w*/*v*% gel concentration. (**c**,**f**): The IR of KDP crystals grown in solution, normal KDP crystals grown in gel, and kebab-like KDP crystals grown in gel. The dotted lines are used to indicate the peak positions of the vibration modes of δ(P-O-H⋯O) and v(PO_4_) in the IR spectra. (**g**) Methanol, (**h**) ethanol, (**i**) isopropanol as an antisolvent, and OM images of KDP crystals obtained in a gel system with a silica gel concentration of 8 *w*/*v*%.

Therefore, we used infrared (IR) spectroscopy to verify interactions between the silica gel and the KDP prismatic crystal faces. Figure 4c presents the IR of KDP crystals grown under different conditions in the range of 800–1400 cm^−1^. The peak around 1280 cm^−1^ is attributed to δ(P-O-H…O), corresponding to the bending vibrations of the P-O-H group and hydrogen bond interactions. The characteristic peaks in the range of 800–1200 cm^−1^ are associated with ν(PO_4_), representing the stretching vibration of the phosphate groups [40]. As shown in Figure 4f, the peak positions of normal KDP crystals grown in the gel exhibit a slight redshift and peak broadening, indicating that the gel network participates in the hydrogen-bonding framework formed by the P-O-H groups on the KDP prismatic face. Following the alteration of gel conditions that resulted in the growth of kebab-like crystals, the peak positions of the KDP crystals shifted further to the red. They broadened, indicating that the interaction between the gel network and the hydrogen bonds formed by the P-O-H groups on the KDP prismatic face has intensified. This suggests that, in a silica gel medium, the hydroxyl groups on the gel network can interact with the phosphate groups on the prismatic face, resulting in a similar effect to that of an “impurity stopper”. Additionally, by modifying the gel’s internal conditions, the interactions can be strengthened, leading to a greater difference in growth rates between the prismatic and pyramidal faces.

Additionally, the gel medium can fix the preformed crystal in situ and continue to inhibit the convection and the mass transport [42]. After the preformation of the needle-like crystal, the gel medium generates a concentration gradient, remaining undisturbed by density-driven convection or crystal settling [43,44]. With the further diffusion of the antisolvent (methanol), the supersaturation of KDP in the system continued to increase. However, the lower KDP concentration hindered the primary nucleation; hence, the secondary nucleation occurred on the surface of needle-like crystals. And the lack of KDP makes the secondary nucleated crystals unable to grow as long as the preformed one. As a result, multiple KDP crystals were lined up along a needle-like crystal, which we called the “kebab-like KDP crystal”.

**Figure A2:**
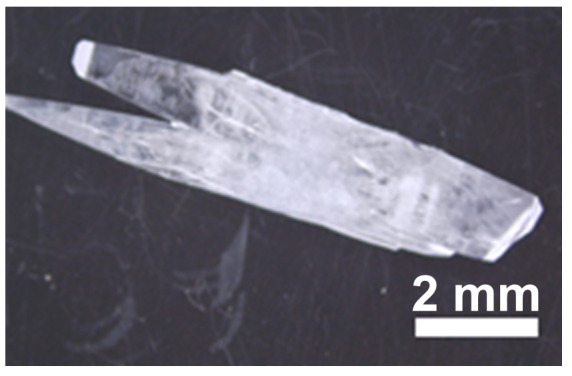
KDP crystals were obtained by cooling crystallization in 30 *w*/*v*% KDP and 1 *w*/*v*% silica sol (α 3.0).

When the concentration of sodium metasilicate was low in the KDP-saturated solution, it was configured as a sol system. By cooling crystallization, the aspect ratio of KDP crystals increased significantly (Figure A2) compared with that of the pure solution (Figure 1b). It indicates that the hydroxyl groups on the silica are sufficient to affect the growth rate of KDP crystal planes.

### 2.2. The Influence of HAc Concentration

The experiments in the solution system (Figure 3g–i) indicated that only increasing HAc concentration cannot lead to the formation of kebab-like KDP crystals. Therefore, the synergetic effect between the HAc and silica gel is the critical factor in forming the kebab-like KDP crystals.

In the same silica gel, by increasing the HAc concentration, after hydrolysis and condensation of sodium metasilicate, more uncondensed hydroxyl groups would be exposed on the interface of the silica gel networks. Thermogravimetric analysis experiments showed that with the increase in HAc concentration, the amount of hydroxyl groups in the silica gel increased (Figure 4a,b,d,e). As shown in Figure 4a,b, when the temperature is below 200 °C, silica gel loses physical and chemical adsorption water to produce the weight loss peak. And when it is above 200 °C, the hydroxyl groups in the silica gel will condense and dehydrate to produce another weight loss peak [45,46]. Thus, a higher HAc concentration produces more pronounced differences between the growth rates of prismatic and pyramidal crystal faces, leading to the formation of a high-aspect-ratio KDP crystal (needle-like) and giving rise to a further kebab-like crystal appearance.

### 2.3. The Influence of Antisolvent

The formation mechanism of kebab-like crystals was further explored by changing the antisolvent. Using methanol, ethanol, or isopropyl alcohol as an antisolvent led to different growth phenomena in the silica gel system with the same gel formula. The decisive difference between these three antisolvents is the diffusion rate in the silica gel. Using ethanol, which diffused more slowly than methanol in silica gel, only a small number of kebab-like crystals appeared (Figure 4h). For the slowest-diffusing isopropyl alcohol, there were no kebab-like crystals in the system (Figure 4i). Methanol diffused into the gel in the gas phase and supported crystal growth in approximately 1–2 days. In comparison, ethanol took about 4–5 days for the crystals to develop, while isopropanol, being the slowest, required more than 7 days. These differences primarily stem from variations in their boiling points, vapor pressures, and molecular diffusion ability. The slower diffusion rates of antisolvents slow down the growth rate of crystals in the gel system, reducing the growth rate difference between pyramidal and prismatic. Therefore, the growth of the critical needle-like crystals is inhibited, which greatly reduces the appearance of kebab-like crystals.

## 3. Materials and Methods

Chemicals: Sodium metasilicate pentahydrate (Na_2_SiO_3_·5H_2_O, Aladdin, ≥95.0%, Shanghai, China), potassium dihydrogen phosphate (SCR, ≥99.5%, Shanghai, China), acetic acid (Macklin, HPLC, Shanghai, China), methanol (SCR, AR, Shanghai, China), ethanol (SCR, GR, Shanghai, China), and isopropanol (SCR, AR, Shanghai, China) were used.

Gel preparation: Sodium metasilicate pentahydrate was dissolved in deionized water. To catalyze the condensation, KDP was dissolved in an aqueous acetic acid solution. The sodium metasilicate solution was then added dropwise into the KDP–acetic acid solution using a dropping funnel while stirring magnetically for 20 min. Subsequently, 5 mL of the resulting sol was transferred into a 20 mL cylindrical bottle (25 mm in diameter and 60 mm in height). All solutions were then left until gelling (overnight), after which the pH of the gel was measured.

Crystal growth: After gelation in the previous step, 6 mL of methanol was carefully added on top of the gel, followed by inverted growth at 20 °C. Methanol diffused into the gel through the gas phase. The contents of KDP and acetic acid in different groups are shown in Table 1. After two days, KDP crystals appeared in the gel. Then, the solution in the bottle was poured out, the KDP crystal was taken out of the gel, and it was fully washed with methanol. Finally, the crystals were dried in an oven at 60 °C for 20 min. There are two ways to crystallize in solution. The first is cooling crystallization. A 30 *w*/*v*% KDP solution is configured at 40 °C and cooled to 20 °C for slow crystallization. The other is antisolvent crystallization, through the gas phase diffusion of methanol, so that the KDP in the solution slowly crystallizes.

Crystal characterization: Crystal morphology was imaged with an optical microscope (Leica DM 2500 P, Wetzlar, Germany) equipped with a digital camera and SEM (HITACHI, Tokyo, S-4800, 20 kV, Japan). The phase composition of the crystals was determined by powder X-ray diffraction (RIGAKU D/MAX 2550 PC, Tokyo, Japan), and the unit cell parameters of the crystals were obtained by single-crystal X-ray diffraction (Bruker D8 Venture Ims3.0, Karlsruhe, Germany). The Si concentration of the grown crystals in the gel was determined by an inductively coupled plasma mass spectrometer (ICP-MS, PerkinElmer, NexION 300Q, Waltham, MA, USA). The silica gel hydroxyl content was investigated by a simultaneous TGA analyzer (TA, TGA55, New Castle, DE, USA) in air at a heating rate of 10 °C/min. The IR of KDP crystals was obtained by grinding them into a powder and then analyzing them using an IR spectrometer (Thermo Scientific, Waltham, MA, USA).

## 4. Conclusions

In summary, the mechanism of the influence of hydroxyl groups on the gel network on the growth morphology of KDP crystals is proposed. The results show that in the silica gel, the hydroxyl groups on the silica gel networks will form hydrogen bond interactions with the phosphoric acid groups of the KDP crystals. This interaction leads to the “attraction” of the gels to the growth surface, resulting in the hindered growth of the crystal prismatic face and the rapid growth of the pyramidal face, finally forming needle-like crystals with a high aspect ratio. And due to the characteristics of crystal growth in the gel, the needle-like crystal is fixed in situ, and with the increase in supersaturation, secondary nucleation occurs on it to form a kebab-like structure.

## Figures and Tables

**Table 1:** Optimal design of crystal growth conditions ^1^.

No.	C Gel (*w*/*v*%)	KDP (g)	HAc (%) ^2^	HAc (mL)	Kebab-Like Crystal ^3^
a	4.0	5.5	100 (α = 1)	1.30	×
b	4.0	5.5	200 (α = 2)	2.60	√
c	4.0	5.5	300 (α = 3)	3.90	√
d	7.0	5.5	200 (α = 2)	4.50	×
e	7.0	5.5	300 (α = 3)	6.80	√
f	7.0	5.5	400 (α = 4)	9.05	√

^1^ The total volume used was 60 mL. ^2^ The concentration of 100% acetic acid signifies that its stoichiometric ratio is sufficient to completely react with sodium metasilicate. In this case, the amount of acetic acid is twice that of sodium metasilicate, which has the same significance as α = 1. ^3^ × indicates the absence of kebab-like crystal growth, while √ indicates the presence of kebab-like crystal growth.

**Figure A1:**
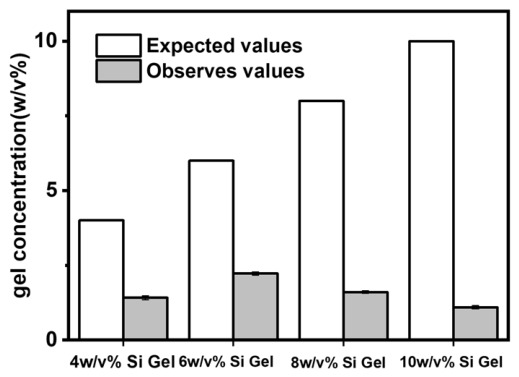
Silica gel content in KDP crystals grown in different concentrations of silica gel.

**Figure A3:**
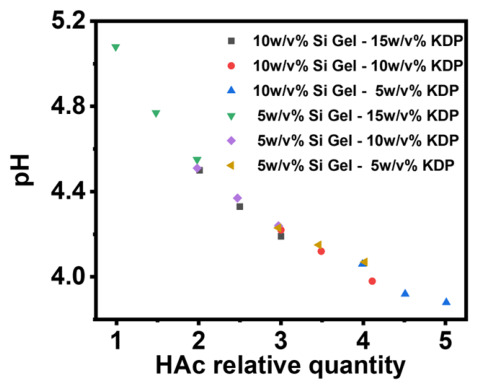
Changes in pH of the system with different gel concentrations, KDP concentrations, and relative additions of acetic acid.

## Data Availability

The original contributions presented in this study are included in the article. Further inquiries can be directed to the corresponding authors.

## Appendix A

Through ICP testing, it was found that as the gel concentration increased, the content incorporated in the kebab-like KDP crystals showed a trend of first increasing and then decreasing (Figure A1). The reason for this change may be attributed to the changes in gel strength and crystallization speed caused by the increase in gel concentration.
